# Dietary Regulation of the Crosstalk between Gut Microbiome and Immune Response in Inflammatory Bowel Disease

**DOI:** 10.3390/foods10020368

**Published:** 2021-02-08

**Authors:** Qianqian Yao, Huiying Li, Linlin Fan, Yangdong Zhang, Shengguo Zhao, Nan Zheng, Jiaqi Wang

**Affiliations:** 1 Key Laboratory of Quality & Safety Control for Milk and Dairy Products of Ministry of Agriculture and Rural Affairs, Institute of Animal Sciences, Chinese Academy of Agricultural Sciences, Beijing 100193, China; qqyao130@gmail.com (Q.Y.); lihuiying@caas.cn (H.L.); fxxfll0825@gmail.com (L.F.); zhangyangdong@caas.cn (Y.Z.); zhaoshengguo@caas.cn (S.Z.); zhengnan@caas.cn (N.Z.); 2 Laboratory of Quality and Safety Risk Assessment for Dairy Products of Ministry of Agriculture and Rural Affairs, Institute of Animal Sciences, Chinese Academy of Agricultural Sciences, Beijing 100193, China; 3 State Key Laboratory of Animal Nutrition, Institute of Animal Sciences, Chinese Academy of Agricultural Sciences, Beijing 100193, China

**Keywords:** inflammatory bowel disease, microbiome, dysbiosis, diet nutrients, immune response

## Abstract

Inflammatory bowel disease (IBD), a chronic, recurring inflammatory response, is a growing global public health issue. It results from the aberrant crosstalk among environmental factors, gut microbiota, the immune system, and host genetics, with microbiota serving as the core of communication for differently-sourced signals. In the susceptible host, dysbiosis, characterized by the bloom of facultative anaerobic bacteria and the decline of community diversity and balance, can trigger an aberrant immune response that leads to reduced tolerance against commensal microbiota. In IBD, such dysbiosis has been profoundly proven in animal models, as well as clinic data analysis; however, it has not yet been conclusively ascertained whether dysbiosis actually promotes the disease or is simply a consequence of the inflammatory disorder. Better insight into the complex network of interactions between food, the intestinal microbiome, and host immune response will, therefore, contribute significantly to the diagnosis, treatment, and management of IBD. In this article, we review the ways in which the mutualistic circle of dietary nutrients, gut microbiota, and the immune system becomes anomalous during the IBD process, and discuss the roles of bacterial factors in shaping the intestinal inflammatory barrier and adjusting immune capacity.

## 1. Introduction

Inflammatory bowel disease (IBD), which is characterized by chronic and relapsing intestinal inflammatory response, and which is also classified as ulcerative colitis (UC) and Crohn’s disease (CD) depending on the clinical manifestations, is growing in prevalence across the world. The pathogenesis of IBD involves mainly susceptible and perturbed interactions between environmental factors, gut commensal microbiota, and the host immune response; however, the specific pathogenesis involved therein is still unclear. Recently, a growing number of studies have focused on the microbial changes that occur during IBD, in attempts to explore new and more efficient approaches to its treatment and management.

The human intestinal tract, particularly the colon, harbors as many as 1000 known species of microbiota, most of which are commensal bacteria [1,2]. Commensal bacteria are of great significance for their host in terms of nutrition and immunity, assisting in the metabolization of dietary nutrients into short-chain fatty acids (SCFAs), amino acids, and vitamins to support the intestinal immune barrier by providing energy substrates for the intestinal epithelial structure. Furthermore, they promote the maturation and cultivation of the immune system, resisting pathogenic bacteria colonization to help maintain homeostasis in the intestinal environment [3,4,5]. In IBD, however, drastic and widespread taxonomic and functional alternations of the microbiota occur, including the enrichment of microbiome-mediated cell signal pathways [6,7,8] and harmful microbiome-produced metabolites [9,10]. Due to its frontline exposure to external environmental factors, the gut microbiota is constantly impacted by a range of dietary nutrients, which are made increasingly complex by modern food-processing technologies and the addition of chemical preservatives. Consequently, it is essential that the roles played by dietary nutrients and fermentation by the intestinal microbiome in the onset of IBD are urgently explored [11].

This article reviews the current literature on the interactions of nutrients in the average daily diet, intestinal microbiota, and host immune responses in IBD, and discusses the possibility of strategically manipulating microbiota metabolisms and composition during gut inflammation through the consumption of specific substrates.

## 2. Intestinal Microbiota and IBD

### 2.1. The Prevalence of IBD Worldwide 

**Figure 1:**
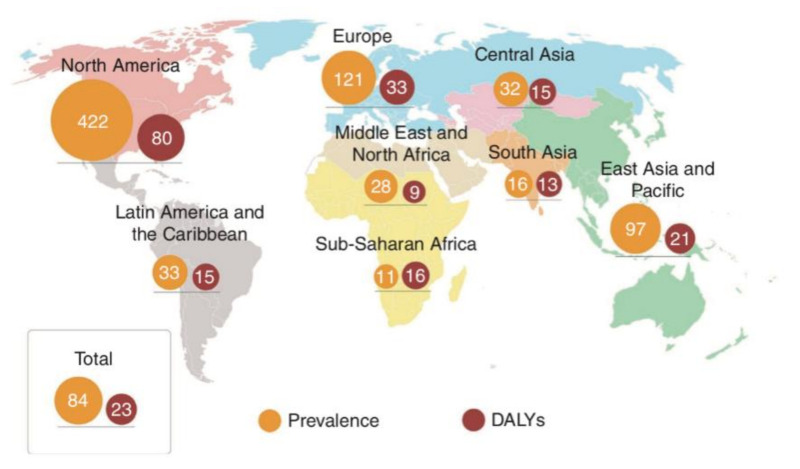
Age-standardized estimates of the regional and global prevalence of IBD (per 100,000 population), as expressed by 2017 disability-adjusted life year (DALY) rates (per 100,000 person-years) [16].

Although IBD can occur in people of any age, from infants to octogenarians, it is diagnosed most often during adolescence and early adulthood [12]. The rising prevalence of the disorder has made it a global public health problem, with enormous associated costs (Figure 1). In Canada, more than 200,000 individuals were diagnosed with IBD in 2012, with direct medical costs amounting to more than CDN $1.2 billion [13]. In Europe, there are currently 2.5 to 3 million patients with IBD, with an estimated annual direct medical cost of €4.6 to 5.6 billion [13,14]. It has been estimated that, by 2025, the number of IBD cases in Western countries will reach 0.5% of the total population, resulting in a correspondingly dramatic increase in the risk of IBD-related complications, such as colon cancer, coronary artery disease, and osteoporosis [15]. Although the incidence of IBD in China is lower than that in Western countries, it has been increasing since 2010. Statistical data indicate that IBD cases are more prevalent in Western developed countries than in emerging countries. This phenomenon has been linked to the changes in lifestyle behaviors (such as decreased physical activity, and increased levels of stress) and diet (a reduction in fiber intake and fat-feeding) experienced by urban dwellers, where among those, diet may play a crucial role [12,16,17,18,19].

### 2.2. Dietary Nutrients and IBD: A Complex Interaction

As a source of luminal antigens, dietary nutrients are thought to be an important factor in the immunopathogenesis of IBD. Although the contribution of diet composition to IBD has long been assumed, it has been scientifically assessed only in retrospective studies [16,20], which are prone to recall bias. In this article, we review the most recent evidence of the risk factors involved in dietary constituents in the development of IBD.

### 2.3. Dietary Fat

**Figure 2:**
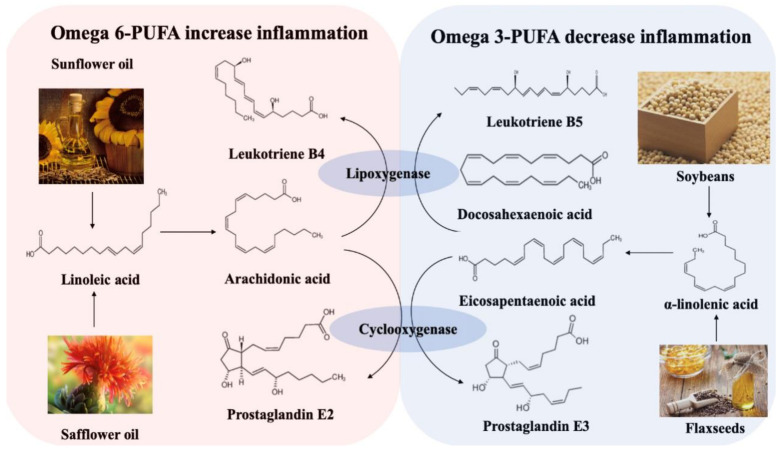
Metabolism of omega-6 and omega-3 polyunsaturated fatty acids [26].

Foods that are high in saturated and trans fats, but low in mono- and polyunsaturated fats, are known to induce numerous health problems, yet they remain largely characteristic of the modern Western diet. Two large prospective studies exploring the relationship between dietary fatty acids and IBD were conducted in Europe [21,22], one of which, from EPIC Study Investigators, showed that a high intake of linoleic acid (LA) increases the risk of UC. LA is an omega-6 polyunsaturated fatty acid (PUFA) prevalent in red meat and cooking oils. It can be metabolized to arachidonic acid (AA) in colonocyte membranes. Such membrane-released AAs generate multiple pro-inflammatory factors, such as prostaglandin E2, leukotriene B4, and thromboxane A2 [23,24,25], all of which are considered to exaggerate the inflammation response in IBD. However, α-linoleic acid (ALA), an omega-3 PUFA, can be metabolized into docosahexaenoic acid (DHA) and eicosapentaenoic acid (EPA), and then further degraded to leukotriene B5 and prostaglandin E3, which help to decrease inflammation. Omega-3 and omega-6 PUFAs competitively utilize the lipoxygenase and cyclooxygenase in the metabolic process, with increases in the availability of omega-3 PUFAs inhibiting the metabolism of omega-6 PUFA. The increases of omega-6 PUFA will, in turn, inhibit the omega-3 PUFAs metabolism (Figure 2).

In a systemic review, Hou et al. [22] evaluated the association between diet and IBD using data from 2609 IBD patients (1269 CD and 1340 UC patients) and over 4000 controls within guideline-recommended methodology, and reported a positive association between the high intake of total fats, PUFAs, omega-6 fatty acids, and meat, and the incidence of CD and UC. In a clinical trial, Bamba et al. [27] showed that fatty acid composition could regulate the anti-inflammatory effects of diets. In that study, CD patients received defined-formula diets with different fat contents over a period of 4 weeks, after which remission rates of 80%, 40%, and 25% were reported in the low-, medium-, and high-fat groups, respectively. Nevertheless, other researchers have proposed a different view on the connection between n-3 PUFA, inflammation, and IBD. Thies et al. [28] reported that a daily supplement of 700 mg AA over a period of three months did not significantly increase pro-inflammatory cytokines in vivo. Thus, it is considered controversial to emphasize the significance of limiting n-6 PUFA, while simultaneously increasing n-3 intake. Instead, the proper ratio of n-6: n-3 PUFA may be deemed more reasonable and persuasive for the prevention and intervention of IBD. Moreover, most previously reported clinical results were based on adult data and, therefore, cannot be directly extended to younger age groups, children, or infants. 

### 2.4. Dietary Fiber 

In the intestine, short-chain fatty acids (SCFAs), including mainly acetic acid, propionic acid, and butyric acid, are generated by the gut microbiota fermentation of dietary fiber. They serve as an energy source for enterocytes and reinforce the intestinal barrier. In one study, a trinitro-benzene sulfonic acid (TNBS)-induced rat colitis model was constructed, and a fiber-supplemented diet containing 5% *Plantago ovata* seeds was administered for two weeks before TNBS induction, then maintained for another week. The fiber-supplemented diet was found to exert anti-inflammation activity that remitted CD by decreasing the production of tumor necrosis factor α (TNF-α), lowering nitric oxide synthase (NOS) activity and elevating the production of SCFA (mainly butyric acid) [29]. In a meta-analysis including two cohort studies, one nested-control study and five case-control studies conducted to quantitatively summarize the evidence regarding dietary fiber and IBD, it was found that a linear dose-response relationship existed between dietary fiber and CD incidence, and that a daily fiber intake of 10 g/d could decrease the risk of CD by 13% [30]. This benefit may rely mostly on the lumen butyric acid produced by dietary fiber, which can regulate gut microbiota composition, maintain immunological homeostasis, and decrease colonic permeability through multiple metabolic pathways [31,32,33]. These results suggest that fiber-rich foods or supplements could be an adjunct to pharmacological treatment. Moreover, dietary fiber has been found to precisely modulate the microbiome to produce SCFAs. A study found that chemically-modified resistant starches with small structural differences (maize-, potato-, and tapioca-derived type IV resistant starches) induced a divergent but highly specific effect on intestinal flora and directly changed the production of propionic acid or butyric acid [34]. In the future, targeted changes in the metabolic and immunological relationship between gut microbiota and the human host can be expected through the alteration of the ratio of SCFAs [35], which may become a novel method through which to provide protection from various intestine-related diseases.

### 2.5. Protein

As one of the main nutritional elements in the human diet, protein provides essential amino acids, both to build new tissues for growth and reproduction, and to repair worn tissues. However, studies in animal models and clinical cases have demonstrated that protein intake is also associated with IBD [22,36]. A large prospective cohort, comprising a group of 40- to 65-year-old women living in France, evaluated the role of dietary proteins in the etiology of IBD and demonstrated that animal proteins derived from meat or fish, but not including eggs or dairy products, could significantly increase the incidence of IBD [37]. A positive correlation between the risk of IBD and animal protein intake was also observed in an investigation that tracked the health of CD patients for more than 20 years [38]. These results suggest that not only the quantity, but also the sources of protein may be associated with IBD; yet, in contrast, another study found that protein intake had no correlation to the development of IBD [39]. Due to the complexity and variety of diets and the problems associated with the collection of reliable dietary data, the impact of protein intake on IBD risk has not been studied sufficiently to reach a persuasive conclusion. However, despite the limited research findings on the impact of a high-protein diet on IBD, the effects of such a diet on intestinal homeostasis, particularly in the colonic microbiome and mucosa, suggest that it may influence the pathogenesis of IBD. 

### 2.6. Vitamin D

Vitamin D, whether synthesized in the skin or absorbed via eggs, fatty fish, or dairy products, undergoes two hydroxylation steps to become biologically active 1,25-dihydroxyvitamin D (1,25(OH)_2_D) in the body [40]. It is primarily known to regulate the bone metabolism by controlling intestinal calcium absorption [41], but it has also become increasingly apparent that vitamin D participates in a variety of other diseases, including IBD [42]. A retrospective study involving 3217 patients (55% CD, mean age 49 years) identified plasma 25-hydroxy vitamin D [25(OH)D] ≥ 30 ng/mL as sufficient. Moreover, this study showed that in CD patients, plasma 25(OH)D < 20 ng/mL was more commonly associated with IBD-related hospitalization than in those with sufficient levels of 25(OH)D [43]. Similar estimates were also seen in patients with UC. Furthermore, CD patients who had initial levels <30 ng/mL, but who subsequently normalized their 25(OH)D, had a reduced likelihood of surgery compared to those who remained deficient [43]. In another cross-sectional study of 182 CD patients and 62 healthy controls, active CD was found to be associated with low serum 25(OH)D levels [44]. To evaluate the association of serum vitamin D and the course of the disease over a five-year follow-up, 965 IBD patients (598 CD, 367 UC) were recruited, of which 29.9% showed low mean vitamin D levels [45]. Low levels of vitamin D have also been associated with higher morbidity and disease severity, indicating the potential importance of monitoring and treating deficiency. Vitamin D has also been found to influence innate immunity by acting directly on the T cell to enhance Th2 cell development [46,47], as well as CD4 + T differentiation into Th17 cells [48]. Such results may, at least in part, support the assumption of vitamin D’s protective role in autoimmune diseases, and more research is required to explore its specific roles in IBD.

### 2.7. Other Nutrients

Given the oxidative stress involved in IBD pathogenesis [49,50], trace elements have also been considered to impact the disease. Zinc, an important trace element present in body fluids and tissues, is known to play a pivotal role in wound repair and tissue regeneration. A study comprising 995 patients with IBD (773 CD patients, 223 UC patients) found that zinc deficiency was positively correlated with an increased risk of surgery- and disease-related complications, while normalization of zinc levels was associated with improvements in these outcomes in both CD and UC patients [51]. Another retrospective chart reviewed patients diagnosed with IBD (ages 1 to 18) and showed that zinc deficiency was common in patients with newly diagnosed IBD [52]. The study further recommended that zinc levels should be assessed at the time of diagnosis, so that enteral repletion may commence in cases of deficiency [52]. Zinc had been also found to regulate the inflammation process and antioxidant effects via the activation of Nf-κB and SOD1 signaling pathways, by which it might impact IBD pathogenesis [53,54,55]. Despite these evidently crucial roles of zinc, the status of this trace element in IBD has not been conclusively researched; however, it has been widely asserted that zinc supplements may be effective in its treatment.

Accumulating evidence indicates that the consumption of excess amounts of sugar can increase the risks of diabetes [56], coronary heart disease [57], and other chronic diseases [58]. Sugar raises the serum levels of total cholesterol, low-density lipoprotein (LDL) and HDL, and blood pressure, even within isocaloric replacement and in the absence of weight gain [59], and has been proven to affect the development of IBD. Maaz et al. [60] verified the relationship between sugar consumption and IBD pathogenesis in their study of 859 IBD patients over a two-year period, in which high sugar intake was found to increase inflammatory biomarkers and reduce their quality of life. Significantly, the question regarding the proper sugar intake for people with IBD has not yet been addressed. It also should be considered that modern food processing methods may further influence the effects of sugar in the body.

### 2.8. Dietary Nutrients and Microbiota

Among the environmental factors involved in the etiology of IBD, diet is considered to be most significant, due to its flexible manipulation and alteration of the composition of gut microbiota (Table 1). There is substantial evidence to suggest that our gut microbiota has profound effects on our health, and that dysbiosis can lead to multiple health problems, including inflammation, obesity, and mood disorders. Consequently, growing knowledge of the close relationship between diet and gut microbiota suggests that an oriented shift of microbiota composition, through the consumption of a specific diet, may present a novel approach to improving health or even reversing diseases, including IBD.

### 2.9. Gut Microbiota and IBD: Dysbiosis Is a Typical Feature in IBD

Statistical data analysis has shown that the effects of diet on IBD vary from person to person, mainly because of the diversity of gut microflora [69,70,71,72]. Our intestines are home to a wide variety of microorganisms, some still unknown. Protein, sugar, and fiber in our diets can be metabolized into nutrients by digestive enzymes secreted by commensal bacteria, which are then absorbed by intestinal epithelial cells or circulated in the blood to every corner of the body. IBD patients tend to have reduced microbial diversity and richness of gut microbiota [73,74]. This is referred to as dysbiosis, and it can lead to an intestinal disorder, further affecting the absorption of nutrients. Indeed, numerous studies based on patients and murine models have proved the crucial role of the microbiome in IBD, although the issue of whether dysbiosis is causative or consequential in the onset of inflammation remains controversial [75,76,77]. 

As shown in Table 2, the composition of microbiota shows oriented changes among IBD patients with a decreased abundance of *Firmicutes*, and increased *Escherichia coli* and *Campylobacter concisus* [78,79,80,81,82,83,84,85]. A study conducted by Joossens et al. [78] explored the compositional changes in predominantly fecal microbiota via the analysis of 207 fecal samples from CD patients and matched heathy individuals. Decreases were found in *Dialister invisus*, an uncharacterized species of *Clostridium* cluster XIVa, *Faecalibacterium prausnitzii* and *Bifidobacterium adolescentis*, while an increase in *Ruminococcus gnavus* was observed in comparison to the healthy controls. *Faecalibacterium prausnitzii* is one of the favorable commensal species in the gut, belonging to the phyla *Firmicutes*. It has been reported to promote a healthy intestinal environment by increasing butyrate production and lowering oxygen tension [86], which indicates that this bacterium plays an important role in the anti-inflammation process in hosts. Moreover, the unbalance between *Firmicutes* and *Bacteroidetes* is a distinctive sign in IBD, and known to promote inflammation [87,88,89].

In contrast, increased concentrations of *Proteobacteria*, particularly *Escherichia coli*, have been documented in both mucosa-associated and fecal samples of CD patients, in comparison to controls [90,91,92]. CD-associated *Escherichia coli*, namely adhesion-invasive *Escherichia coli* (AIEC), has been observed to exert pro-inflammatory properties [93]. Compared with non-inflamed controls, mucosa-associated *Escherichia coli* was found to be more commonly present in the colonic biopsy samples of CD patients (79%), among which AIEC accounted for 53% [94]. A higher concentration of AIEC strains could be cultured from the ileal mucosa of Crohn’s ileitis patients compared to colonic CD patients [95,96]; however, the abundance of AIEC was not as high in UC patients as it was in the CD individuals [97]. AIEC adherence to the intestinal epithelium increases the permeability between intestinal epithelial cells and, consequently, the invasion of pathogenic bacteria to lamina propria, which induces inflammation in susceptible hosts [98]. Due to the diverse serology of AIEC and its phylotypes, as well as the limited investigation of its virulence-associated features [98], it is difficult to correlate it with CD deterioration. Moreover, the mechanism by which AIEC survive within phagocytic cells is unclear and should be investigated.

*Mycobacterium avium* subspecies paratuberculosis (MAP) is an obligate intracellular pathogen causing spontaneous granulomatous enterocolitis in ruminants. Peripheral blood samples were collected from 202 IBD patients, 24 non-IBD controls, and 29 healthy individuals for nested PCR and 16S rRNA seqencing IS900-specific nested PCR [84]. It was found that active CD patients had the highest MAP DNA prevalence among IBD patients (68%); however, these levels were found to decrease after infliximab treatment, suggesting facilitative mechanisms between the host and MAP in CD pathogenesis [84]. Another paper also observed the positive relationship between MAP and CD patients, 87% of whom were detected to carry MAP DNA [99]. MAP infection occurs widely in farm animals, especially dairy herds [100], and it is difficult to eliminate by pasteurization, which is a cause for human concern [101]. Based on current findings, however, it is difficult to draw a firm conclusion about the relationship between MAP and IBD. Further research is required to uncover the mechanism through which it may be involved in IBD pathogenesis.

### 2.10. Gut Microbiome Dysbiosis Induces IBD: Role of Immunological Barrier 

The intestinal tract is a digestion and absorption organ, serving also as a barrier to prevent the invasion of foreign antigens and pathogens. The intestine is an extremely complex system, comprising various physical, chemical, microbiological, and immunological barriers. The biological barrier of the intestine comprises mainly commensal gut microbiota, able to harvest energy from polysaccharides and to resist the invasion of pathogens for their host through the secretion of antimicrobial peptides and via competitive colonization [102,103,104]. When pathogens escape these chemical and physical barriers, the intestinal epithelial cells stimulate the expression of inflammatory factors and chemokines, which respond to external stimuli through corresponding signaling pathways, while simultaneously recruiting more white blood cells to kill and clear damaged cells or pathogens. However, changes in the structure or function of this intestinal barrier system can increase the risk of infection, bacterial translocation, and bacterial imbalance, thus decreasing the host’s overall health [105,106]. Recent studies have, therefore, investigated how the interactions between gut microbiota and the immune system influence the development of IBD. It is proposed that a compromised mucosal immune function, including an increase in intestinal permeability or epithelial cell injury, may enable an abnormally high concentration of pathogens to be transmitted to underlying lamina propria, consequently triggering a persistent inflammation response in genetically susceptible individuals [107]. 

### 2.11. Microbiota and Intestinal Epithelial Cells

Intestinal epithelial cells, including absorbent cells, goblet cells, Paneth cells, M cells, and undifferentiated cells, form the first line of defense against the invasion of enteric pathogens. This barrier facilitates the selective absorption of nutrients, while blocking the transmission of pathogens to the lamina propria through the apical–junctional complex (AJC) and tight junctions (TJs) between intestinal epithelial cells [108,109]. Under normal conditions, the physical barrier controls the transcellular and paracellular permeation of antigens and allows only small molecules to cross into the mucosa, which are subsequently eliminated by host immune cells. However, under pathological conditions, occludin and immunoglobulin expressions are decreased, leading to increased permeability and, consequently, permitting increased numbers of bacterial antigens to migrate to the mucosa, ultimately inducing inflammation [110,111]. Intestinal epithelial cells express pattern recognition receptors (PRRs) to combine with pathogen-associated molecular patterns (PAMP) on the surface of pathogenic microorganisms, thus exerting the recognition of microbial antigens. The cell-surface membrane glycoproteins and glycolipids of the intestinal epithelium are thought to serve as a bridge between host and gut microbiomes. *Faecalibacterium prausnitzii*, a commensal bacterium, produces a 15 kDa protein. This protein was found to exert an anti-inflammation effect by inhibiting the NF-κB pathway in intestinal epithelial cells to prevent colitis in a DNBS-induced colitis mice model. Moreover, the transfection of this protein cDNA into epithelial cell lines significantly decreased the activation of the NF-κB pathway in a dose-dependent manner [112]. L-fucose is one of the most abundant surface molecules in intestinal epithelial cells. Segmented filamentous bacteria (SFB), a Bacteroides species, decorate surface capsular polysaccharides and glycoproteins with L-fucose, protecting the intestinal tract from pathogens by promoting the colonization of intestinal symbiotic microbiota and inhibiting pathogens. In addition, the advantage of SFB colonization of the intestine under competitive conditions is lost in the SFB mutant [113]. 

### 2.12. Migration of Mucosal Bacteria in IBD 

The intestinal mucus layer, consisting mainly of mucin 2 (MUC2), prevents direct interactions between commensal bacterial and epithelium cells. In the colon, the mucus is divided into two layers, with the outer layer commonly colonized by gut bacteria and the inner layer attached to intestinal epithelium cells to separate bacteria. The majority of bacteria interact indirectly with intestinal epithelial cells, mediated by food-fermented postbiotics [114,115]. The mucosa also serves as a source of nutrients for commensal bacteria, such as *Akkermansia muciniphila*, while changes in the microbiome community, mainly the ratio of *Bacteroides* to *Firmicutes*, could alter mucin glycosylation [116,117,118]. In a heathy status, gut microbiota is restricted to the outer layer of mucosa, stimulating active B cells to secrete secretory IgA (sIgA), while promoting goblet cells to produce MUC2 [119]. Under stress conditions, however, such as trauma, infection, and shock, sIgA secretion is decreased, thus increasing the chance of bacterial migration to the submucosa and leading to inflammation and mucosal barrier injury [120]. Swidsinski et al. [121] investigated the mucosal flora community of colonic biopsies from bowel inflammation patients (305) and controls (40) and reported a thick bacterial band attached to the mucosa, indicating that the function of the mucosal barrier to hold back bacteria may be seriously disturbed in IBD. Moreover, the increase in mucosal bacterial species can either drive or inhibit certain bacteria, which may, in turn, exaggerate inflammation. Thus, the modification of the types or numbers of bacteria present in the mucosa may provide an effective alternative method of IBD treatment (Table 3). 

## 3. GALT Dysfunction during IBD

The gut contains several complex and diverse immune regions, the most important of which is the gut-associated lymphoid tissue (GALT), as it represents the intestinal frontier of the systemic immune response [129]. GALT consists of lymphoid tissue and lymphocytes distributed throughout the lumen, with the former including Peyer’s patches (PP), isolated lymph follicles (ILF), and mesenteric lymph nodes (MLN), while the latter is composed mainly of intraepithelial lymphocytes (IEL) and lamina propria lymphocytes (LPL) in intestinal lamina propria [130]. These structures contain different types and proportions of immune cells, such as B cells, T cells, mononuclear macrophages, mast cells, dendritic cells, and granulocytes, which together play crucial roles in the host immune system [131,132]. As GALT is responsible for the recognition and neutralization of harmful antigens, it is critical in whether a response is one of inflammation or tolerance. GALT either maintains a low reactive immune surveillance state or activates immune tolerance mechanisms for harmless signal stimuli, including the induction of systemic immune tolerance and sIgA secretion. However, it responds quickly against hazard signals by inducing inflammation, thus aiding in resistance against invasion by pathogenic microorganisms and maintaining the stability of the intestinal environment [116,133,134].

In the submucosa, immune cells act as a second line of host defense, promoting tissue regeneration in cases of injury. When arriving at the lamina propria, antigens from foods or microorganisms are presented to T cells via processing by antigen-presenting cells (APC), subsequently activating either CD8+ or CD4+ T cells, the two of which exert opposite effects. CD8+ cells can kill pathogen-infected cells and downregulate the immune system. CD4+ T cells are differentiated into four types, namely Th1, Th2, regulatory T cells (Treg), and Th17 cells, each with different functions under different circumstances [135,136,137]. Th1 cells secrete IFN-γ and participate in cell-mediated immune responses. Under the induction of IL-4, CD4+ T cells differentiate into Th2 cells, secreting IL-4, IL-5, and IL-13, and are involved in humoral immune responses. Treg cells can release TGF-β, CD25, and forkhead box protein 3 (FoxP3) to participate in immune regulation [131,138], while TGF-β and IL-6 co-induce CD4+ T cells to differentiate into Th17, which is involved in inflammatory responses and autoimmune diseases [139]. Th17 cells could prevent the colonization of pathogens in the intestine by secreting the cytokines interleukin (IL-17A, IL-17F, and IL-22), thereby activating the production of antimicrobial proteins in intestinal epithelial cells and reinforcing intercellular tight junctions [140]. Th17 cells are induced after SFB adheres to the intestinal epithelium, driving the release of IgA and production of pIgR [141,142,143]. Treg cells are classified as either thymus-derived or peripherally derived. The former is responsible for recognizing self-antigens and regulating the autoreactive T-cell function, while the latter identifies microbial antigens and controls the tolerance to non-self-antigens [144].

Hence, the intestinal barrier is comprised of structural components (mucus and epithelial cells), immune cells (intraepithelial and submucosal immune cells), and soluble agents (IgA and antimicrobial peptides), which respond to microorganisms [145]. Any sharp or dramatic changes to the system can alter the intestinal barrier, probably inducing inflammation. The intestinal microbiome is thought to be involved in altering the intestinal barrier by activating intestinal immune cells and epithelial cells to secrete various cytokines, leading to local intestinal and systemic immune responses during IBD. 

## 4. Microbiome and GALT

**Figure 3:**
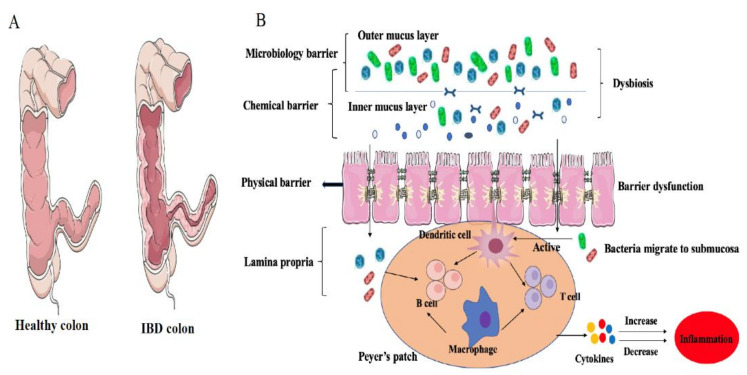
The physical state of the intestinal immune system in IBD hosts: (**A**) Comparison between the intestinal structures of a healthy colon and a colon with Crohn’s disease; (**B**) In IBD, a cascade inflammatory reaction happened, including colonic dysbiosis, enteric epithelial barrier dysfunction, active immunocyte, increased levels of cytokines.

Gut symbiotic bacteria are a functional requirement for GALT. In turn, dysbiosis and the invasion of pathogens trigger aberrant immune responses, leading to local or systemic inflammation (Figure 3). Disruption of the Thl/Th2 ratio is considered an indication of IBD pathogenesis [146]. In a study of sterile mice, when the numbers of intestinal Thl and Thl7 decreased, the intestinal immune response was controlled by Th2, while this imbalance of Thl/Th2 could be improved through the colonization of *Bacteroides fragilis*, indicating that intestinal microbiome colonization plays an important role in regulating imbalance in the immune response of T cells, thus mitigating the onset of IBD [147]. In addition, polysaccharide A produced by *Bacteroides fragilis* was found to exert anti-inflammation activity via the suppression of pro-inflammatory IL-17 and an increase in the production of IL-10 secretion by CD4+ T cells [148]. In the GALT of autoimmune arthritis, the inflammatory products released by gut commensal bacteria dynamically enhanced the antigenic responsiveness of T cells, leading to a more serious inflammatory response [149]. Several specific strains, belonging to IV, XIVa, and XVIII of *Clostridia*, which were isolated from healthy human feces, were found to expand and promote Treg cells differentiation, and oral administration of those strains attenuated the inflammatory responses in adult mice models of colitis [150]. Moreover, the interactions of surface layer proteins A (SlpA) of *Lactobacillus acidophilus* with SIGNR3 (specific intracellular adhesion molecule-3 grabbing non-integrin homolog-related 3) were found to mitigate colitis by maintaining the balance of intestinal microbiota and protecting the mucosal barrier [151]. All the above results suggest that manipulation of the gut microbiota might present a potential and novel approach for the treatment of inflammatory diseases. Despite the discovery of a large number of probiotics, there remains an urgent need for in-depth exploration of those microbes that can induce a stronger therapeutic response, are host-compatible, and can affect specific branches of the host immune system in a well-controlled manner. 

## 5. Conclusions and Perspectives

A better understanding of the complicated relationships between food, the intestinal microbiome, and host immune response is of crucial importance in defining the onset of IBD. The combination of daily diet nutrients and pathogenic invasion contributes significantly to dysbiosis, which is a typical characteristic of IBD. Numerous therapies have been presented for the treatment of IBD, including probiotics, antibiotics, and specific diets, each with varying results. Most recently, a new approach involving fecal microbiota transplantation from the gut microbiota of a healthy host to that of the diseased host, has proven effective and worthy of further investigation.

**Figure 4:**
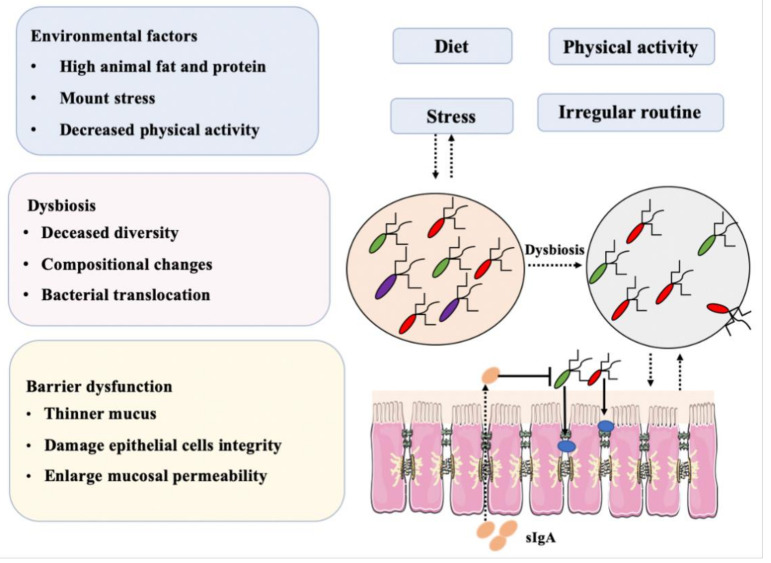
Combination of factors, mainly diet and dysbiosis, that may induce IBD.

In the study of intestinal bacteria, it is necessary to approach the human body holistically, as an extremely complex ecosystem (Figure 4). Bacteria and cells, as key elements in this system, mutually influence each other’s actions as they participate in the various physiological activities within our body. Any external stimulus could, eventually, induce multiple cascade reactions and, therefore, in examining the intestinal microbiome or microbiome-related diseases, no cell, organ or bacterium should be viewed in isolation if the ultimate goal is to ensure the good health of the entire system.

## Figures and Tables

**Table 1:** Effects of diet on gut microbiota.

Dietary Strategy	Characteristics	Affected Species	References
Gluten-free diet	No gluten	*Bifidobacterium* *↓* *Lactobacillus* *↓* *Enterobacteriaceae* *↑* *Escherichia coli* *↑* *Prevotella* *↓*	[61,62,63]
Mediterranean diet	High fiber, low red meat	*Prevotella* ↑ *Lachnospira* ↑ *Bifidobacteria* ↑ *Lactobacillus* ↑ *Bacteroides* ↑ *Clostridium* ↓	[64,65,66]
Western diet	High animal fat, high animal protein	*Bifidobacteria ↓* *Lactobacillus↓* *Bacteroides* ↑ *Enterobacteria* ↑	[67,68]

**Table 2:** Microbiome alterations during inflammatory bowel disease (IBD).

Samples	Type of Disease	Increased	Decreased	References
Fecal samples of 68 CD patients, 84 of their unaffected relatives and 55 matched healthy individuals	CD	*Ruminococcus gnavus*	*Dialister invisus, Faecalibacterium prausnitzii, Bifidobacterium adolescentis*	[78]
190 tissue colon samples from CD, UC, and non-IBD control	UC, UD	*Bacillus*, *Proteobacteria*, *Actinobacteria*	*Firmicutes and Bacteroidetes*, *Lachnospiraceae*	[79]
18 fecal samples from active UC patients and healthy control	UC	Active *Escherichia coli*	Biodiversity of active bacteria	[80]
Fecal samples from CD patients (*n* = 161) and healthy individuals (*n* = 121)	CD	*Bacteroides, Prevotella, Proteus*	*Faecalibacterium, Fusobacterium, Eubacterium, Bifidobacterium,*	[81]
8 samples from active colonic CD patients and 16 from healthy volunteers	CD	*Escherichia coli,* microflora diversity	*Clostridium coccoides, Bacteroides*	[82]
Biopsies from 5 different locations between ileum and rectum in 10 twin pairs	CD	*Escherichia coli*	*F. prausnitzii* (in ileal CD)	[83]
Peripheral blood from 202 IBD patients, 24 non-IBD controls and 29 healthy individuals	IBD	*Mycobacterium avium* subsp. *paratuberculosis,* adherent-invasive *Escherichia coli*	No detection	[84]
301 biopsies from between ileum and rectum of 15 CD, 13 UC and 33 healthy individuals	CD, UC	*Campylobacter concisus*	No detection	[85]

**Table 3:** Bacteria-mediated changes in the mucosal barrier in IBD.

Animal Model/Clinic Data Analysis	Mucosal Bacteria	Outcome	References
345 colonic biopsies samples from bowel inflammation patients (305) and controls (40)	High concentrations of bacteria attached to mucosa in patients, no translocation	Mucosal bacteria increased with the severity of inflammation	[121]
Biopsy specimens from 72 UC, 12 CD patients and 65 healthy controls	Harmful bacterial groups increased, while beneficial bacterial species declined	Components of mucosal flora changed in IBD patients	[122]
MDCK1 cells infected with *Campylobacter jejuni*	—	Monolayer integrity changed, affected tight junction protein ZO-1 distribution	[123]
Mucosal biopsies from CD, UC, disease and healthy controls (*n* = 63)	Active mucosa-attached microbiota changed in IBD patients	Altered inflammation status	[124]
120 biopsies from controls (20), self-limiting colitis (SLC, 20), UC (20) and randomly individual (60)	Bacteria found within mucus in UC, SLC	Bacterial migration, adherence to and invasion of the mucosa	[125]
Mucosal and submucosal samples from CD and controls	4 and 13 bacterial species were found within submucosa at the center and margin of disease	Changed bacteria might drive or inhibit certain organisms in CD	[126]
35 ilea mucosal and submucosal tissues from CD patients (*n* = 20) and healthy controls (*n* = 15)	*Ruminococcus* spp., *Oscillospira* spp., *Pseudobutyrivibrio* spp., and *Tumebacillus spp* increased in subjacent submucosa	Bacterial migration to submucosal resulting from mucosal barrier injury	[127]
Intestinal biopsies from IBD patients (inflammation, non-inflammation) and from controls	Streptococcus spp. accounted for 80% in the inflamed mucosa of CD; mucus layer in the inflamed IBD patients was remarkably thinner	*Streptococcus* spp. increased with the severity of IBD	[128]
